# Characterization of the complete chloroplast genome sequences of six *Dalbergia* species and its comparative analysis in the subfamily of Papilionoideae (Fabaceae)

**DOI:** 10.7717/peerj.13570

**Published:** 2022-07-01

**Authors:** Changhong Li, Yu Liu, Furong Lin, Yongqi Zheng, Ping Huang

**Affiliations:** 1 State Key Laboratory of Tree Genetics and Breeding, Research Institute of Forestry, Chinese Academy of Forestry, Beijing, China; 2 Key Laboratory of Resource Plant Innovation and Utilization, Institute of Subtropical Crops of Zhejiang Province, Zhejiang Academy of Agricultural Sciences, Wenzhou, Zhejiang, China

**Keywords:** *Dalbergia* genus, Chloroplast genome, Sequence diversity, Gene selective pressure, Phylogenetic relationship

## Abstract

*Dalbergia* spp. are numerous and widely distributed in pantropical areas in Asia, Africa and America, and most of the species have important economic and ecological value as precious timber. In this study, we determined and characterized six complete chloroplast genomes of *Dalbergia* species (*Dalbergia obtusifolia*, *D. hupeana*, *D. mimosoides*, *D. sissoo*, *D. hancei*, *D. balansae*), which displayed the typical quadripartite structure of angiosperms. The sizes of the genomes ranged from 155,698 bp (*D. hancei*) to 156,419 bp (*D. obtusifolia*). The complete chloroplast genomes of *Dalbergia* include 37 tRNA genes, eight rRNA genes and 84 protein-coding genes. We analysed the sequence diversity of *Dalberigia* chloroplast genomes coupled with previous reports. The results showed 12 noncoding regions (*rps16-accD*, *trnR-UCU-trnG-UCC*, *ndhE-ndhG*, *trnG-UCC-psbZ*, *rps8-rpl14*, *trnP-UGG-psaJ*, *ndhH-rps15*, *trnQ-UUG-rps16*, *trnS-GCU-psbI*, *rps12-clpP*, *psbA-trnK-UUU*, t*rnK-UUU-intron)*, and four coding regions (*rps16*, *ycf1*, *rps15* and *ndhF*) showed many nucleotide variations that could be used as potential molecular markers. Based on a site-specific model, we analysed the selective pressure of chloroplast genes in *Dalbergia* species. Twenty-two genes with positively selected sites were detected, involving the photosynthetic system (*ndhC*, *adhD*, *ndhF*, *petB*, *psaA*, *psaB*,* psbB*, *psbC*,* psbK* and *rbcL*), self-replication category of genes (*rpoA*, *rpoC2*, *rps3*,* rps12* and *rps18*) and others (*accD*, *ccsA*, *cemA*, *clpP*, *matK*, *ycf1 and ycf2*). Additionally, we identified potential RNA editing sites that were relatively conserved in the genus *Dalbergia*. Furthermore, the comparative analysis of cp genomes of Dalbergieae species indicated that the boundary of IRs/SSC was highly variable, which resulted in the size variation of cp genomes. Finally, phylogenetic analysis showed an inferred phylogenetic tree of Papilionoideae species with high bootstrap support and suggested that Amorpheae was the sister of the clade Dalbergieae. Moreover, three genera of the *Pterocarpus* clade showed a nested evolutionary relationship. These complete cp genomes provided valuable information for understanding the genetic variation and phylogenetic relationship of *Dalbergia* species with their relatives.

## Introduction

Chloroplasts (cp) play a vital role in the photosynthetic cells of plants and algae. They contain a double-stranded genome with an independent replicated system, and it is generally recognized that chloroplasts may evolve from cyanobacteria through endosymbiosis (Daniell et al., 2016; Mcfadden & Dooren, 2004). The structure of the cp genome is relatively conserved in plants, including two small inverted repeats (IRa and IRb), a long single-copy (LSC) region and a short single-copy (SSC) region (Palmer & Jeffrey, 1985). The size of the cp genome ranges from 107 kb to 218 kb. However, a variety of mutations have occurred in the cp genome during a long-term evolutionary process, which can help us understand how plants evolved. Therefore, the mutation in the cp genome is used for the study of evolution, phylogeny and phytogeography in plants (Sancho et al., 2017; Yang et al., 2013).

The genus *Dalbergia* belongs to the Fabaceae family and includes over 250 species distributed in pantropical areas in Asia, Africa and America (Klitgaard & Lavin, 2005). Many *Dalbergia* species are known as high-value rosewood because of their decorative and excellent wood quality, and their heartwoods with a wide range of colour variation have been used to produce luxury furniture and musical instruments (Bhagwat et al., 2015). Therefore, these species have been suffering from fast-evolving threats that have led to a major decline in wild populations (Winfield, Grayson & Scott, 2016). Aiming to enhance the worldwide protection of rosewood species, an updated list of *Dalbergia* species has been adopted by CITES (Convention on International Trade in Endangered Species of Wild Fauna and Flora) since 2017, which will support better protection for the diversity and health of rosewood populations (Hung et al., 2020). A complete infrageneric classification of the *Dalbergia* genus has been built by Bentham (George & Bentham, 1860), but a wide range of species and distribution makes it difficult to perform a comprehensive investigation (Carvalho, 1997; Chen, Zhang & Larsen, 2010; Niyomdham, 2002; Prain, 1904; Sunarno & Ohashi, 1997; Thothathri, 1987; Win, 2020). The first molecular phylogenetic framework of the *Dalbergia* genus has been established based on a limited number of *Dalbergia* species. However, the results suggest that the *Dalbergia* genus is monophyletic and originated from the New World (Vatanparast et al., 2013). Cui proposed a superior and comprehensive phylogenetic framework of the *Dalbergia* genus based on morphological traits, chloroplast and *ITS* DNA sequences, and more extensive samples (Cui, 2014; Li, 2017). Due to the higher posterior probability or bootstrap percentages of a phylogenomic tree, it could provide new insight into phylogenetic relationships among different taxa. In addition, a large number of cp genomes of *Dalbergia* and its relative taxa in Papilionoideae have been published in GenBank in recent years (Wariss et al., 2018; Deng et al., 2018; Xu et al., 2019; Liu et al., 2019; Song et al., 2019; Qin et al., 2020; Hong et al., 2020; Win et al., 2020). These published cp genomes may give us an opportunity to improve previous phylogenetic frameworks. Besides that, more genetic variation features from cp genome could provide new insight to understand the evolution and adaptation of *Dalbergia* plants in heterogeneous habitats, such as selective pressure and RNA editing site of chloroplast genes. RNA editing is the posttranscriptional modification of an RNA nucleotide sequence to produce more variation of transcripts. Previous reports suggest it may not only be involved in early evolution of land plants, but also be considered to be as a communication mechanism between chloroplast and nucleus to acclimatize a changing world (Schallenberg-Rüdinger & Knoop, 2016; Zhao, Huang & Chory, 2019).

In this article, six complete cp genomes of *Dalbergia* species (four tree species and two vine species) were de novo assembled by next-generation sequencing, and comparative genomic analysis of *Dalbergia* species and its related species in the subfamily of Papilionoideae (Fabaceae) was performed. The study aims to (1) obtain a few complete cp genomes of *Dalbergia* species; (2) reveal the cp genomic characteristics of *Dalbergia* species; (3) compare the genomic features of Dalbergieae species; and (4) establish an inferred phylogenetic relationship of Papilionoideae species.

## Materials & Methods

### Sample collections and DNA extraction

Fresh healthy leaves were collected from seedlings of three *Dalbergia* species (*D. obtusifolia*, *D. hupeana and D. sissoo*) in the greenhouse of the Research Institute of Forestry in the Chinese Academy of Forestry in Beijing (116°14′25″, 40°0′14″). In addition, leaf samples of *D. balansae* were collected in Yinjiang, Tongren, Guizhou (108°22′41″, 28°0′44″), and leaf samples of *D. hancei* were collected from Shiqian, Tongren, Guizhou (108°17′12″, 27°31′05″). The leaf samples of *D. mimosoides* were collected from Puer, Yunnan (100°36′01″, 22°35′03″). The voucher specimen was deposited in the Research Institute of Forestry, Chinese Academy of Forestry in Beijing, China. Genomic DNA was isolated by a modified CTAB method (Doyle, 1987), and agarose gel electrophoresis and a one-drop spectrophotometer were used to detect DNA integrity and quality (OD-1000, Shanghai Cytoeasy Biotech Co., Ltd., Shanghai, China).

### DNA sequencing, genome assembly and validation

We constructed shotgun libraries (150 bp) with genomic DNA and sequenced them on the BGI-500 platform. High-quality clean data were obtained by trimming the original data from both ends and removing the adapter and low-quality reads. Following quality filtering, we employed Bowtie2 v2.2.6 to map reads on a local cp genome database (Langmead & Salzberg, 2012). NOVOPlasty and CAP3 were used to de novo assemble the cp genomes with the starting sequence (Huang & Madan, 1999; Nicolas, Patrick & Guillaume, 2017). We used Blast, Hmmer 3, Aragorn and manual correction to predict gene, rRNA and tRNA sequences (Altschul et al. 1997; Dean & Bjorn, 2004; Eddy & Pearson, 2011). Organellar GenomeDRAW v1.3.1 was employed to draw a circular map of the cp genome, and CGV95 was adopted to visualize the annotation results (Lohse, Drechsel & Bock, 2007). Then, we adopted 34 primer pairs to prove junctions in six *Dalbergia* species cp genomes by PCR-based sequencing. A 20- µL reaction volume was set as the PCR program with a thermal cycler (Applied Biosystems, Foster, CA, USA): 2 ×Es Taq MasterMix (Dye) (CWbio, Beijing, China) with 10 µL, DNA with approximately 50 ng, forward primer and reverse primer with 5 pmol, respectively, and sterile double-distilled water was added to the 20 µL volume. We employed the following procedure to perform PCR amplification: 5 min of denaturation at 94 °C; 94 °C with 35 cycles of denaturation for 30 s, the optimal temperature to anneal for 30 s, followed by 30 s with 72 °C for extension; and then 5 min at 72 °C in the amplifications eventually for extension. After PCR amplification was completed, the amplified products were sequenced and compared with the assembled chloroplast genome (Table S1). Eventually, these accurate chloroplast genomes were submitted and stored in the NCBI GenBank (https://www.ncbi.nlm.nih.gov/, accession numbers MN714219–714222, MN905599 and MN833948). These newly assembled cp genomes of the *Dalbergia* genus were analysed for codon usage patterns. The protein-coding genes with more than 300 nucleotides were extracted to analyse the codon usage indices, including the relative synonymous codon usage (RSCU) and codon adaptation index (CAI), by using CodonW v1.4.4 (http://codonw.Sourceforge.net/). RSCU values can directly reflect codon usage bias. When RSCU values approach 1, all synonymous codons encoding the same amino acid were used equally. CAI refers to the adaptation index of all codons actually encoding the protein for the case where the optimal codon is used to encode the protein. It is also used to measure the level of gene expression. Higher CAI values implied a strong codon usage bias and a higher expression level (Sharp & Li, 1987).

### Repeat sequence analyses in the cp genome

We adopted MIcroSAtellite (http://pgrc.ipk-gatersleben.de/misa/) to analyse simple sequence repeats (SSRs) in the cp genomes. The SSR motif types of mononucleotide, dinucleotide, trinucleotide tetranucleotide, pentanucleotide and hexanucleotide were set as 10, 6, 5, 5, 5, and 5 for the minimum repeat units, respectively, and we designed primer pairs by Primer 3 in the flanking region with all candidate loci (Andreas et al., 2012). We employed the Tandem Repeats Finder to screen tandem repeat sequences. Match -2, Mismatch -7, and Delta-7 were set as the alignment parameters, the minimum alignment score was set at 80, and the maximum period size was set as 500 (Benson, 1999). We used REPuter (https://bibiserv.cebitec.uni-bielefeld.de/reputerl) to analyse palindromic repeat sequences, complement repeats and dispersed repeat sequences, and 30 and 3 were set as the minimum repeat size and maximum base mismatch, respectively (Kurtz & Schleiermacher, 1999).

### Gene selective pressure analysis

The genes under selection were detected by the Muscle (codon) employed in MEGA7 to align the sequences of the protein-coding gene separately in *Dalbergia* (Sudhir, Glen & Koichiro, 2016), and we used Fast-Tree 2.0 to build the maximum likelihood (ML) phylogenetic tree with 1,000 bootstraps based on complete cp genome sequences (Price, Dehal & Arkin, 2010). The site-specific model mainly assumes that different amino acid sites are subject to different selection pressures (regardless of the differences in selection pressure between different branches) in the dataset. This model is mainly employed to detect the effect of positive selection (*ω* > 1) in the CODEML algorithm (Price, Dehal & Arkin, 2010) employed in EasyCodeML (Gao et al., 2019) and consists of seven codon substitution models, containing M0 (one-ratio), M1a (nearly neutral), M2a (positive selection), M3 (discrete), M7 (beta), M8 (*β*& *ω* > 1) and M8a (*β*& *ω* = 1). The likelihood-ratio test was used to compare the fit of these models to the sequence data. Positively selected sites were detected by three site-specific models, M1a *vs.* M2a, M7 *vs.* M8, and M0 *vs.* M3 (Wan-Lin et al., 2018).

### RNA editing sites

RNA editing was first found 30 years ago (Covello & Gray, 1989), and it also occurs within chloroplasts in plants (Small et al., 2020). RNA editing is one of the essential ways to regulate the expression of chloroplast genes at the posttranscriptional level, which causes nucleotide substitutions, deletions and insertions, thereby changing the coding information of the original transcript (Bentolila et al., 2013; Harris, Petersen-Mahrt & Neuberger, 2002). PREP (predictive RNA editor for plants, http://prep.unl.edu/) is an online program for predicting RNA editing sites, with a series of advantages, requiring minimal input, running fast, and high accuracy (Mower, 2009). A total of 35 protein-coding genes from the cp genome of *Dalbergia* species were submitted for predicting potential RNA editing sites with a cut-off value of 0.8.

### Comparative analyses of cp genomes in Dalbergieae species

A total of 37 Dalbergieae species that are available in the NCBI database and six cp genomes of *Dalbergia* species were chosen for comparative genomic analysis. Then, the cp genomes were compared by mVISTA (Frazer et al., 2004) in shuffle-LANGAN mode with default parameters for other options, and *D. obtusifolia* was set as a reference. IRSCOPE was employed to analyse the boundaries between the four main regions of the annotated cp genomes to investigate the contraction or expansion of the IR regions (Ali, Jaakko & Peter, 2018). Noncoding regions (percentage of variability > 25%) and coding regions (percentage of variability > 8%) were selected acted as mutational hotspots in the comparative analyses of *Dalbergia* species. Percentage of variable = (the number of indels + number of nucleotide mutations)/(the length of aligned sites − the length of indels + the number of indels) × 100% was the formula (Dong et al., 2018).

### Phylogenetic analysis

The phylogenetic relationships with the Papilionoideae subfamily members, including 165 cp genomes available in NCBI and six newly assembled genomes of the *Dalbergia* genus, were reconstructed in this study. A set of 77 common genes shared by these cp genomes were aligned by MAFFT v7 with the default settings. Maximum likelihood (ML) and maximum parsimony (MP) were performed to reconstruct the phylogenetic tree. All the gaps were excluded after alignment in two method analyses. ML was used in FastTree 2.0 (Price, Dehal & Arkin, 2010) with 1,000 bootstrap replicates and the GTR model of nucleotide substitution (Kazutaka, Daron & Standley, 2013). PAUP v4.0 (Swofford, 2002) was performed to construct an MP tree with 1,000 ratchet repetitions and a tree bisection-reconnection (TBR) branch swapping algorithm. Phylogenetic trees with bootstrap values (BS) were visualized using ITOL v6 (http://itol.embl.de/) (Letunic & Bork, 2021).

**Figure 1:**
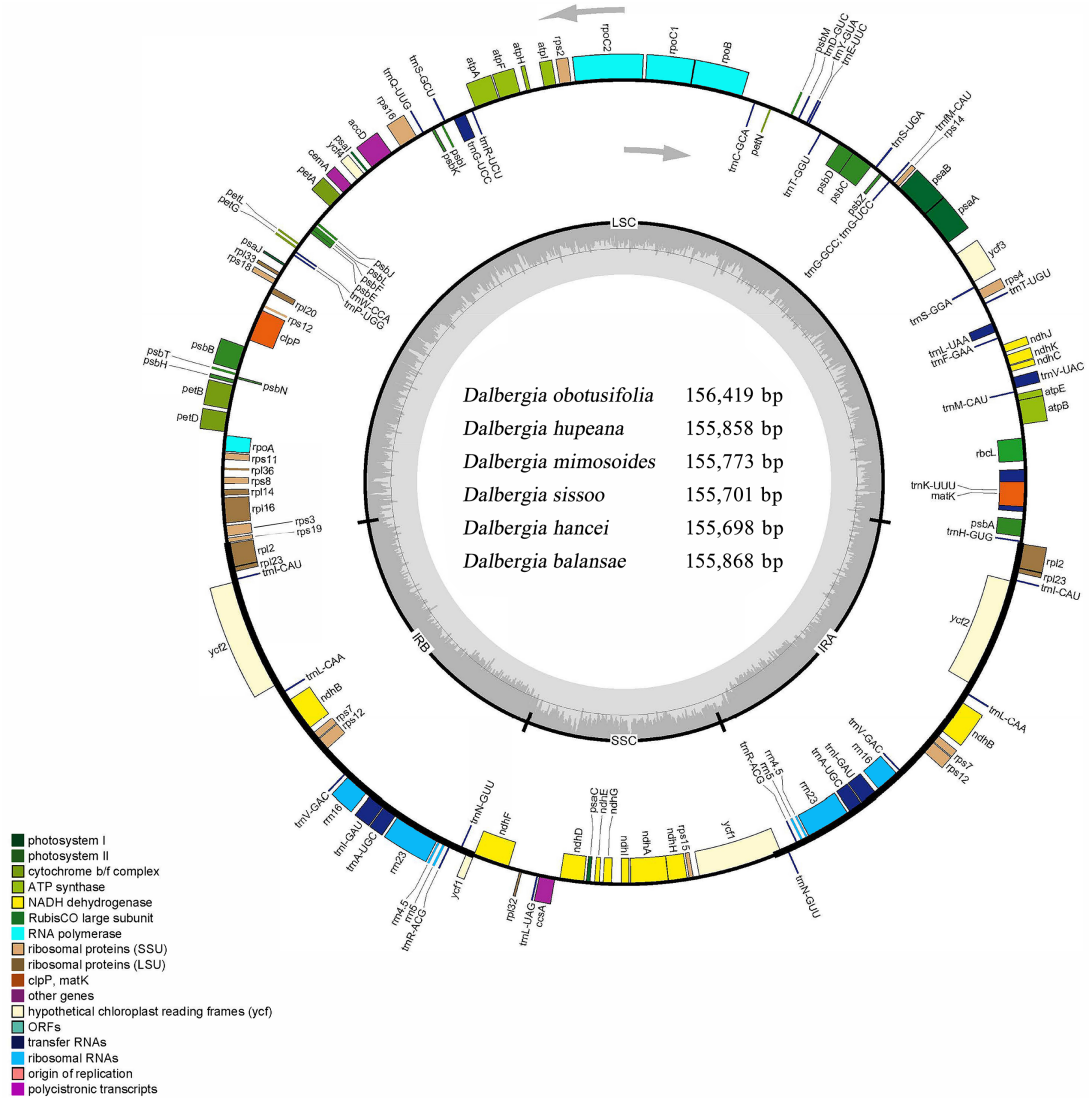
Circular map of the cp genome for *Dalbergia* chloroplast genome. The gray arrows show that genes inside the cycle are transcribed clockwise, and genes outside the circle are transcribed counterclockwise. The innermost shaded areas inside the inner circle correspond to the GC content in the cp genome. Genes in different functional groups are color coded. The boundaries of four regions (IRa, IRb, LSC, SSC) are noted in the inner circle.

**Table 1:** Comparison of features of cp genomes of six *Dalbergia* species.

Taxon	Accession	Genome size (bp)	GC content (%)	LSC (bp)	IRs (bp)	SSC (bp)	Forward repeat(s)	Complement repeat(s)	Reverse repeat(s)	Palindromic repeat(s)	Tandem repeat(s)	SSRs
*D. obtusifolia*	MN714219	156,419	36.00%	85,724	25,699	19,297	32	19	15	40	15	158
*D. hupeana*	MN714220	155,858	36.20%	85,330	25,680	19,168	19	0	6	32	10	156
*D. mimosoides*	MN714221	155,773	36.20%	85,425	25,708	18,932	17	3	3	26	6	151
*D. sissoo*	MN714222	155,701	36.10%	85,387	25,664	18,986	27	4	2	29	12	176
*D. hancei*	MN833948	155,698	36.20%	85,421	25,690	18,897	18	1	0	25	8	149
*D. balansae*	MN905599	155,868	36.20%	85,270	25,672	19,254	19	0	8	31	7	153

## Results

### Chloroplast genome features

Six complete cp genomes of *Dalbergia* spp. ranged from 155,698 bp (*D. hancei*) to 156,419 bp (*D. obtusifolia*), and all the cp genome sequences have been deposited in GenBank (accession Nos. MN714219–714222, MN905599 and MN833948). These cp genomes displayed a typical quadripartite structure consisting of a pair of inverted repeat IR regions (25,664–25,708 bp) separated by one large single-copy (LSC) region (85,270–85,724 bp) and one small single-copy (SSC) region (18,897–19,297 bp) (Fig. 1, Table 1). The overall GC content of the IR region (42.8%) was higher than those of the LSC and SSC regions (approximately 33.7% and 29.5%, respectively) because of the high GC content in rRNAs, which were located in the IR regions. There were 37 tRNA genes, 8 rRNA genes and 84 protein-coding genes in each *Dalbergia* cp genome, and the gene content and order are shown in Fig. 1 and Table 2. Most of these genes did not contain introns, except that 15 genes (*atpF, ndhA, ndhB, petB, petD, rpl2, rpl16, rpoC1, rps16, trnA-UGC, trnG-UCC, trnI-GAU, trnK-UUU, trnL-UAA and trnV-UAC*) contained one intron and three genes (*clpP, ycf3 and rps12*) contained two introns. Thymine (T) and adenine (A) preferences in the third position of the codon were observed in these cp genomes, and codon usage is shown in Table S2.

**Table 2:** List of genes encoded in the cp genomes of the genus *Dalbergia*.

Function	Genes
RNA transfer	trnA-UGC^*^, trnC-GCA,trnD-GUC,trnE-UUC,trnF-GAA,trnfM-CAU, trnG-UCC^*^, trnH-GUG, trnI-CAU, trnI-GAU^*^, trnK-UUU^*^, trnL-CAA, trnL-UAA^*^, trnL-UAG, trnM-CAU ,trnN-GUU, trnP-UGG, trnQ-UUG,trnR-ACG ,trnR-UCU, trnS-GCU, trnS-GGA, trnS-UGA, trnT-GGU, trnT-UGU, trnV-GAC, trnV-UAC ^*^, trnW-CCA, trnY-GUA
RNA ribosomal	rrn16, rrn23, rrn4.5, rrn5
RNA polymerase	rpoC1^*^, rpoC2, rpoA, rpoB
Clp^p^, Matk	clpP^**^, matK
Ribosomal proteins (SSU)	rps2, rps3, rps4, rps7, rps8, rps11, rps12^**^, rps14, rps15, rps16^*^, rps18, rps19
Ribosomal proteins (LSU)	rpl2^*^, rpl14, rpl16^*^, rpl20, rpl23, rpl32, rpl33, rpl36
Hypothetical chloroplast reading frames (ycf)	ycf1, ycf1^Ψ^, ycf2, ycf3^**^, ycf4
ATP synthase	atpA, atpB, atpE, atpF^*^, atpH, atpI
Photosystem I	psaA, psaB, psaC, psaI, psaJ
Photosystem II	psbA, psbB, psbC, psbD, psbE, psbF, psbH, psbI, psbJ, psbK, psbL, psbM, psbN, psbT, psbZ
RubisCO large subunit	rbcL
Cytochrome complex	petA, petB^*^, petD^*^, petG, petL, petN
NADH dehydrogenase	ndhA^*^, ndhB ^*^, ndhC, ndhD ,ndhE, ndhF, ndhG, ndhH, ndhI, ndhJ, ndhK
Others	accD, cemA, ccsA,

**Notes.**

* Genes containing one intron.

** genes containing two introns.

Ψ pseudogene.

### Repeat structure and SSR analyses

We detected repeat structures in the cp genomes of six *Dalbergia* species. The results showed that the cp genome of *D. obtusifolia* contained the largest number of repeat sequences (32 forward, 19 complementary, 15 reverse, 40 palindromic and 15 tandem repeats), and the cp genome of *D. hancei* contained the least number of repeat sequences (18 forward, 1 complementary, 25 palindromic and 8 tandem repeats). Then, reverse repeats were not found in *D. hancei*, and complement repeats were not screened in *D. hupeana* and *D. balansae* (Table 1).

A total of 149–176 candidate cpSSR loci were detected in six *Dalbergia* species (Table 1). A majority of SSR loci were located in the LSC region (72.55%–76.82%), followed by the SSC region (17.88%–22.53%). These findings indicated that the distribution of cpSSRs was imbalanced in the genus *Dalbergia*. Then, mono-, di- and trinucleotide SSRs were detected in each *Dalbergia* species, and the results showed that the majority of the mononucleotide repeats were A or T repeats. The majority of the dinucleotide repeat sequences consisted of AT or TA repeats, while a few TC repeats were also screened (Table S3).

### Selective pressure analysis

The rate of nonsynonymous (dN) and synonymous (dS) substitutions for 77 common protein-coding genes of 21 *Dalbergia* species were compared, and the results showed 22 genes with positive selection sites (Table 3). These genes included one subunit of acetyl-CoA carboxylase gene (*accD*), one subunit of C-type cytochrome synthesis gene (*ccsA*), one subunit of gene code envelope membrane protein (*cemA*), one subunit of cytochrome b/f complex gene (*petB*), one gene of maturase (*matK*), one gene of protease (*clpP*), three NADH-dehydrogenase subunit genes (*ndhC*, *ndhD* and *ndhF*), two subunits of the photosystem I gene (*psaA* and *psaB*), three subunits of the photosystem II gene (*psbB*, *psbC* and *psbK*), one subunit of rubisco gene (*rbcL*), two DNA-dependent RNA polymerase genes (*rpoA* and *rpoC2*), three genes for ribosome small subunit proteins (*rps3*, *rps12* and *rps18*), *ycf1* and *ycf2* genes. According to the M8 model, the ycf1 gene possessed 15 positive sites, followed by *ndhD* (4), *rbcL* (4), *ndhF* (3), ycf2(3), *accD* (2), *matK* (2), *ndhC* (2), *rpoA* (2), and *rpoC2* (2), and the other twelve genes each had only one positive site (*ccsA*, *cemA*, *clpP*, *petB*, *psaA*, *psaB*, *psbB*, *psbC*, *psbK*, *rps3*, *rps12* and *rps18*). In addition, the likelihood ratio tests (LRTs) of variables under different models were compared in the site-specific models, M0 *vs.* M3, M1 *vs.* M2 and M7 *vs.* M8, to support the sites under positive selection (*p* < 0.01) (Table S4).

**Table 3:** Log-likelihood values of the site-specific models, with detected sites having non-synonymous/ synonymous (dN/dS) values >1.

Gene Name	Models	Number of parameters	lnL	Likelihood ratio test *p*-value	Number of positively Selected Site (s)	Positively selected site (s)
accD	M8	45	−2720.664276	0.000000000	2	102 Q 0.978^*^, 496 E 0.963*
	M7	43	−2780.691566			
ccsA	M8	45	−516.193509	0.000000000	1	94 N 0.990^*^
	M7	43	−520.088006			
cemA	M8	45	−1157.782999	0.000000000	1	205 L 0.956^*^
	M7	43	−1219.150432			
clpP	M8	45	−889.260891	0.004273146	1	24 Y 0.997^**^
	M7	43	−894.716296			
matK	M8	45	−2376.612487	0.014212023	2	76 A 0.987^*^, 408 D 0.986*
	M7	43	−2380.866154			
ndhC	M8	45	−594.667603	0.000000000	2	23 L 0.980^*^, 98 L 0.998**
	M7	43	−651.981618			
ndhD	M8	45	−2693.372607	0.000000000	4	220 L 1.000^**^, 299 Q 0.966^*^, 411 Y 0.988^*^, 463 F 0.989*
	M7	43	−2750.835062			
ndhF	M8	45	−3489.422379	0.000283610	3	372 N 0.969^*^, 436 L 0.999^**^, 530 Y 0.994^**^
	M7	43	−3497.590288			
petB	M8	45	−1002.104915	0.000000000	1	204 P 0.976^*^
	M7	43	−1060.388486			
psaA	M8	45	−3497.811392	0.000000000	1	321 E 0.999^**^
	M7	43	−3563.246297			
psaB	M8	45	−3515.015299	0.000000000	1	206 Y 0.999^**^
	M7	43	−3577.728010			
psbB	M8	45	−2306.906597	0.000000000	1	296 Q 0.992**
	M7	43	−2381.411208			
psbC	M8	45	−2252.030367	0.000000000	1	209 I 0.961^*^
	M7	43	−2293.950845			
psbK	M8	45	−277.920155	0.000735872	1	43 V 1.000^**^
	M7	43	−285.134609			
rbcL	M8	45	−2439.780528	0.000000000	4	23 T 0.991^**^, 225 I 1.000^**^, 279 S 1.000^**^, 375 I 0.998^**^
	M7	43	−2508.204270			
rpoA	M8	45	−1665.905867	0.021426153	2	140 T 0.982^*^, 237 F 0.965^*^
	M7	43	−1669.749010			
rpoC2	M8	45	−7455.004187	0.000115194	2	515 L 0.999^**^, 1024 L 0.972^*^
	M7	43	−7464.073079			
rps3	M8	45	−1135.452527	0.000000000	1	66 K 0.999^**^
	M7	43	−1200.963047			
rps12	M8	45	−536.439203	0.014760302	1	116 K 0.973^*^
	M7	43	−540.655017			
rps18	M8	45	−390.409751	0.009407171	1	84 I 0.994^**^
	M7	43	−395.076034			
ycf1	M8	45	−12407.532472	0.000000000	15	118 Q 0.980^*^, 165 L 0.999^**^, 508 S 1.000^**^, 511 Y 0.999^**^, 538 M 0.983^*^, 673 L 0.950^*^ 1004 I 0.996^**^, 1056 P 1.000^**^, 1085 S 0.998^**^, 1092 Q 0.980^*^, 1107 L 0.999^**^, 1138 R 0.981^*^, 1205 D 0.970^*^, 1328 L 0.980^*^, 1508 Q 0.988^*^
	M7	43	−12451.520712		
ycf2	M8	45	−9803.132649	0.000000000	3	660 N 1.000^**^, 933 W 0.995^**^, 1065 S 0.995^**^
	M7	43	−9824.817329			

**Notes.**

* *p* < 0.05.

** *p* < 0.01.

### RNA editing site

We analysed the full set of chloroplast coding sequences, and the results showed that the number of RNA editing sites ranged from 32 to 43 in those six *Dalbergia* species, involving 18–20 protein-coding genes (Table 4). All the predicted RNA editing sites are the conversion of cytosine (C) to thymine (T) and may have caused amino acid changes. A majority of RNA editing occurs in the second codon, and a few occur in the first position of the codon. We also found that the conversion of amino acids caused by RNA editing is mostly from serine (S) to leucine (L). These changes could reverse protein polarity and affect hydrophobicity (Pinard, Myburg & Mizrachi, 2019; Rabah et al., 2017). The results showed that almost half of the RNA editing sites were located in the *ndh* genes; the number of RNA editing sites in the *ndhB* gene was the largest, with 10, followed by *ndhA* (5), *ndhF* (4), *ndhG* (4), *rps2* (3), *accD* (2), *atpI* (2), *ccsA* (2), *matK* (2), *ndhD* (2), *psbL* (2), *rpoA* (2), and *rps16* (2), and the other genes had only one editing site (Table S5). Moreover, we also observed that over half of the conventions at the codon positions changed from S (serine) to L (leucine).

**Table 4:** Predicted RNA editing sites in the cp genomes of *Dalbergia* species.

Taxon	*accD*	*atpF*	*atpI*	*ccsA*	*clpP*	*matK*	*ndhA*	*ndhB*	*ndhD*	*ndhF*	*ndhG*	*psbE*	*psbF*	*psbL*	*rpoA*	*rpoB*	*rpoC1*	*rps14*	*rps16*	*rps2*	total
*D. obtusifolia*	1	1	1	2	1	2	3	9	1	**0**	2	1	1	1	1	1	1	1	2	3	35
*D. hupeana*	1	1	**0**	2	1	2	3	10	2	2	3	1	1	1	1	1	1	1	2	3	39
*D. mimosoides*	1	1	1	2	1	2	3	10	2	2	3	1	1	1	1	1	1	1	2	2	39
*D. sissoo*	1	1	1	2	1	2	3	9	1	**0**	2	1	1	1	1	1	1	1	2	2	34
*D. hancei*	1	1	2	2	1	2	3	9	2	2	3	1	1	1	**0**	1	1	1	2	2	38
*D. balansae*	1	1	**0**	2	1	2	3	9	2	1	3	1	1	1	1	1	1	1	2	3	37
*D. cochinchinensis*	1	1	1	2	1	2	4	9	1	1	2	1	1	2	**0**	1	1	1	1	3	36
*D. oliveri*	2	**0**	1	2	1	2	5	10	2	3	3	1	1	1	1	1	1	1	2	3	43
*D. cultrata*	1	1	1	2	1	2	3	9	2	3	2	1	1	1	1	1	1	1	2	2	38
*D. bariensis*	1	1	1	2	1	2	4	9	2	1	4	1	1	1	1	1	1	1	2	3	40
*D. hainanensis*	1	1	1	2	1	2	4	9	2	1	3	1	1	1	**0**	1	1	1	1	3	37
*D. tonkinensis*	1	1	1	1	1	2	4	9	2	**0**	**0**	1	1	1	1	1	1	1	1	2	32
*D. odorifera*	1	1	1	1	1	2	4	9	**0**	2	2	1	1	1	1	1	1	1	1	2	34
*D. armata*	1	1	1	2	1	2	3	9	2	**0**	2	1	1	1	**0**	1	1	**0**	2	2	33
*D. cearensis*	1	1	1	2	1	2	3	9	1	2	3	1	1	1	**0**	1	1	1	2	2	36
*D. chlorocarpa*	1	1	1	2	1	2	3	8	1	1	2	1	1	1	1	1	1	1	2	2	34
*D. vietnamensis*	1	1	1	1	1	2	4	9	1	2	2	1	1	1	1	1	1	1	2	2	36
*D. frutescens*	1	1	1	2	1	2	3	9	2	4	2	1	1	1	1	1	1	1	2	2	39
*D. martinii*	1	1	1	2	1	2	3	9	1	2	2	1	1	1	2	1	1	1	2	2	37
*D. obovata*	1	1	1	2	1	2	3	9	1	2	2	1	1	1	1	1	1	1	2	2	36
*D. yunnanensis*	1	1	1	1	1	1	4	9	1	1	2	1	1	1	1	1	**0**	1	2	2	33

### Sequence divergence in Dalbergieae species

**Figure 2:**
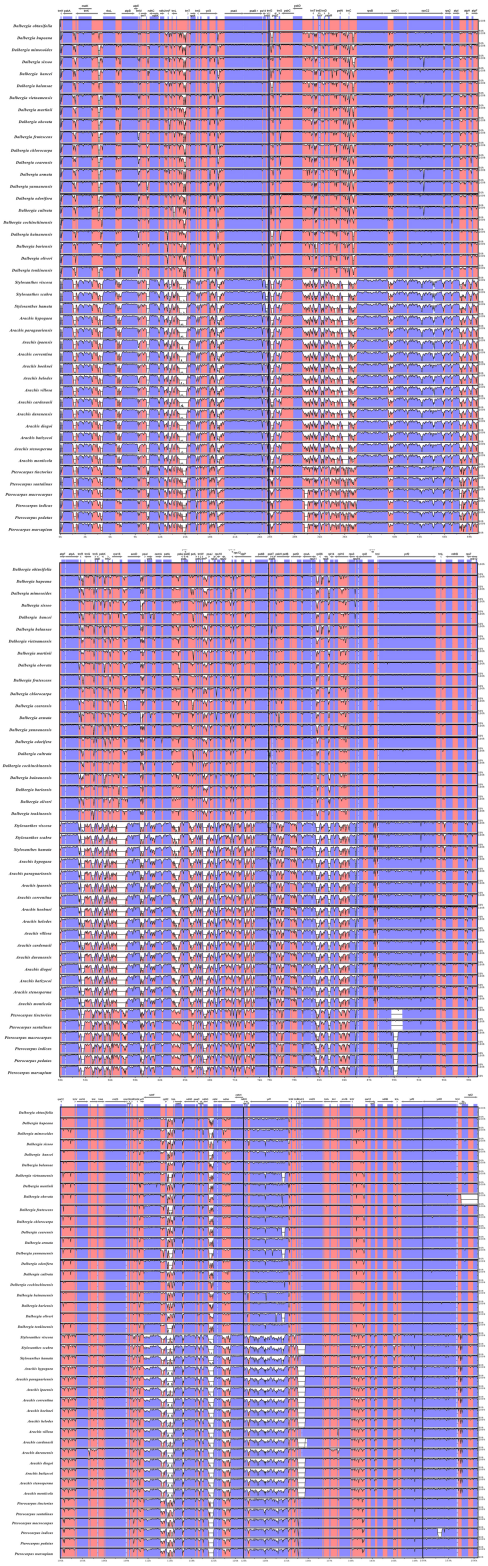
Comparisons of sequence identity of cp genomes for 43 Dalbergieae species. Vertical axis represents identity ranging from 50 to 100%. Each arrow indicates the annotated gene and its transcriptional direction. Genome regions are color coded as an exon, mRNA or tRNA, untranslated region (UTR) and conserved noncoding sequence (CNS).

**Figure 3:**
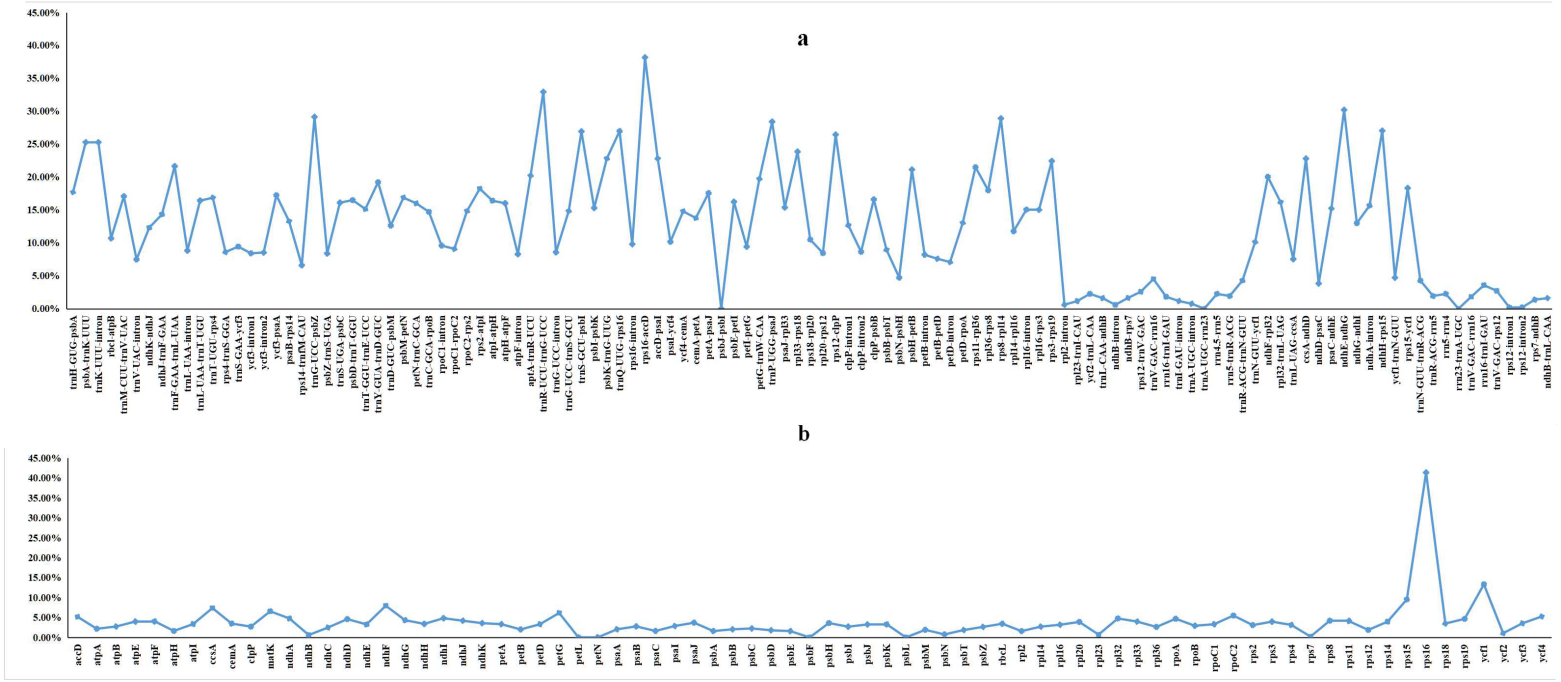
Percentages of variable sites in homologous regions across 21 *Dalbergia* species. (A) The introns and spacers (IGS); and (B) protein coding sequences (CDS).

We performed a BLAST analysis of the complete sequences of 43 cp genomes (21 *Dalbergia* spp., three *Stylosanthes* spp., 13 *Arachis* spp. and 6 *Pterocarpus* spp.) using mVISTA, and the highly divergent regions are shown in Fig. 2; the result showed a high sequence similarity across the cp genomes, with a sequence identity over 70.0%, and the variability of protein-coding regions was less than those of noncoding regions. Twelve regions within the noncoding regions (*rps16-accD*, *trnR-UCU-trnG-UCC*, *ndhE-ndhG*, *trnG-UCC-psbZ*, *rps8-rpl14*, *trnP-UGG-psaJ*, *ndhH-rps15, trnQ-UUG-rps16, trnS-GCU-psbI, rps12-clpP, psbA-trnK-UUU and trnK-UUU-intron*) and 4 regions within the coding regions (*rps16, ycf1, rps15 and ndhF*) showed greater levels of variation (percentage of variability >25% and 8%, respectively) (Fig. 3).

**Figure 4:**
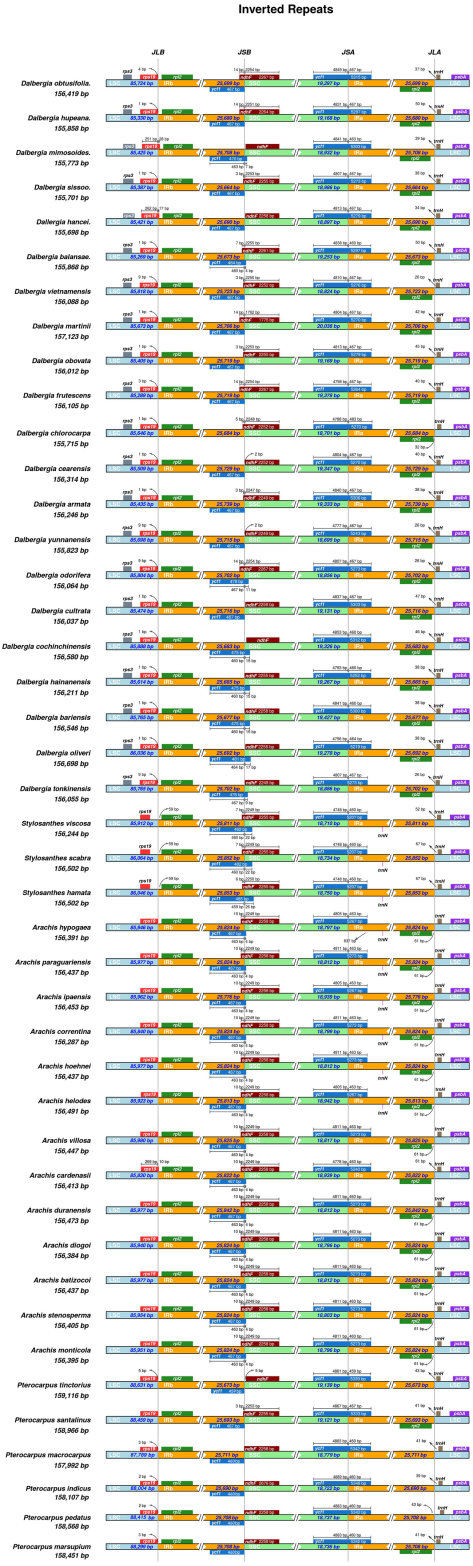
Comparison of the boundaries between LSC, SSC, and two IR regions in cp genomes of Dalbergieae species. The number of base pairs (bp) represents the distance from the boundary to the end of the gene.

The inverted repeat and single-copy (IR/SC) boundary regions of cp genomes were examined and are illustrated in Fig. 4, and the results showed that the boundary of the LSC/IRs was highly conserved, whereas the borders of IRs/SSC were highly variable in Dalbergieae species. First, the *ndhF* gene and *ycf1* pseudogene crossed in the SSC/IRb regions within the two parts for all the *Arachis* and *Stylosanthes* species. Second, the *ycf1* gene was complete in the IRb region for all *Pterocarpus* species and most *Dalbergia* species. Third, the *ycf1* gene crossed the boundary of the SSC/IRa region for all four genera mentioned above.

### Phylogenetic relationship of Papilionoideae species

**Figure 5:**
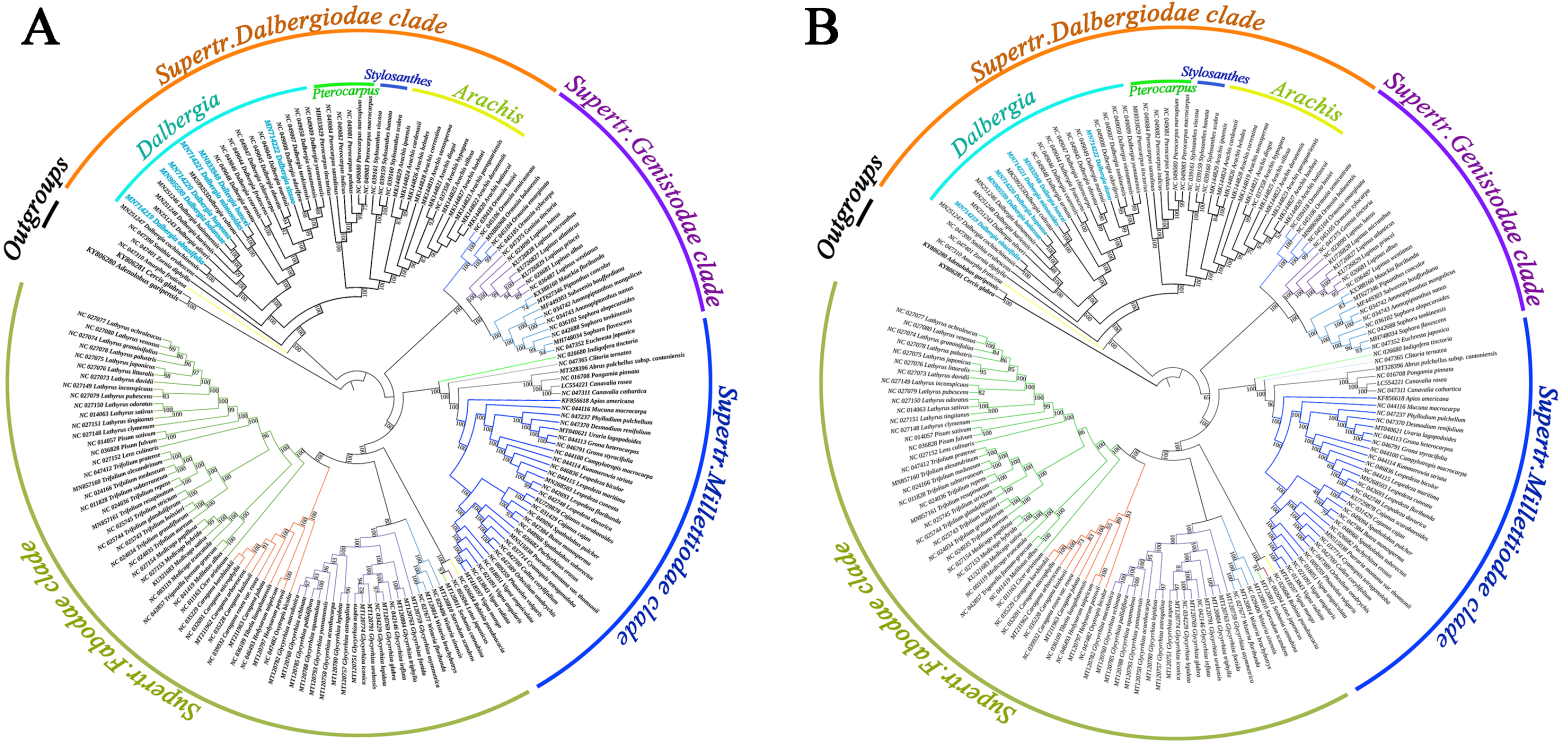
Phylogenetic tree reconstruction of 171 taxa based on 77 genes in the chloroplast genome sequences. (A) Maximum likelihood (ML) method. (B) Maximum parsimony (MP) method.

We screened a total of 77 common genes from the cp genomes of Papilionoideae species to build a phylogenetic tree by performing the maximum likelihood (ML) and maximum parsimony (MP) methods. All the ML and MP trees were highly congruent in identifying these Papilionoideae species of the phylogenetic position. The results showed an inferred phylogenetic tree of Papilionoideae species with high bootstrap support (Fig. 5). Twenty-one species of *Dalbergia* formed a monophyletic group with high support (BS = 100 for ML and MP) and were resolved as an early diverging lineage from the Dalbergieae clade. *Dalbergia* species were clustered into the Supertr. Dalbergiodae with the Amorpheae clade also suggested that Amorpheae was a sister to the Dalbergieae clade. In addition, three genera of the *Pterocarpus* clade (*Pterocarpus*, *Stylosanthes* and *Arachis*) showed a nested evolutionary relationship in the phylogenetic tree.

## Discussion

### Genetic variation in cp genomes

We determined the complete cp genomes of six *Dalbergia* species. These cp genomes have a typical quadripartite structure, and the variation in cp genome sizes for *Dalbergia* species is mainly caused by the sizes of the two single-copy regions. The GC content of SSC regions is lower than that of IR regions, which may result from the presence of rRNA genes in IR regions (Asaf et al., 2016). Similar to most land plants, the protein-coding genes of the cp genome in *Dalbergia* species are highly conserved (Daniell et al., 2016). In addition, we identified over 140 SSRs in six *Dalbergia* species; approximately 70% of the SSRs were distributed in the LSC regions, and the majority of these SSRs were mono- and dinucleotide repeats. SSRs are widely distributed across nuclear and plastid genomes and have a great influence on genome recombination and rearrangement (Guisinger et al., 2010). Additionally, the results suggested that the variation in the boundary of the SC/IR region contributed to the size variation in cp genomes in Dalbergieae species. In the *Dalbergia* species, noncoding regions (percentage of variability > 25%) and coding regions (percentage of variability > 8%) had a large degree of variation and acted as mutational hotspots. The percentage of variation is equal to the sum of indels and the number of nucleotide mutations divided by the length of the alignment site minus the length of indels plus the number of indels, and the final result is multiplied by 100% using the formula (Dong et al., 2018). 12 noncoding regions and 4 coding regions showed greater levels of variation. These sequences could be used to develop potential molecular tools for further study of phylogenetic relationships and population genetics.

### RNA editing sites

RNA editing was first found 30 years ago (Covello & Grey, 1989), and it also occurs within chloroplasts in plants (Small et al., 2020). RNA editing is one of the essential ways to regulate the expression of chloroplast genes at the posttranscriptional level (Bentolila et al., 2013; Harris, Petersen-Mahrt & Neuberger, 2002). RNA editing is a posttranscriptional process that may trigger changes in coding information from original transcripts (Takenaka et al., 2013). Therefore, identifying RNA editing sites in the cp genome will provide us with more information on evolutionary dynamics. The results showed that 12 common genes contained potential RNA editing sites in *Dalbergia* species, accounting for 54.54% of the total. These findings indicated that the variation model of RNA editing sites in cp genomes is relatively conservative for the genus *Dalbergia*. We also found that most of the conversions at the codon could lead to amino acid changes from polar to apolar and could result in an increase in protein hydrophobicity (Pinard, Myburg & Mizrachi, 2019).

### Adaptive evolution of chloroplast genes

We used a site-specific model to estimate the selection pressure, and 22 genes with positive selection sites were found in *Dalbergia* species. The *ycf1* gene is one of the largest genes encoding a part of the chloroplast’s inner envelope membrane protein translocon (Kikuchi et al., 2013), and the *ycf1* gene in the genus *Dalbergia* has been shown to be subject to positive selection from 15 sites. Three NADH dehydrogenase subunit genes (*ndhC, ndhD and ndhF*) possessed at least two positively selected sites, implying that these family members were potentially under positive selective pressure in *Dalbergia* species. NADH-dehydrogenase subunits are important in the electron transport chain for the generation of ATP and are essential components for photosynthesis in plants (Weiss et al., 1991). Additionally, we found that the *rbcL* gene possessed four sites under positive selection. This gene encodes the large subunit of Rubisco protein, which is an important component as a modulator of photosynthetic electron transport (Allahverdiyeva et al., 2005; Piot et al., 2018). The positive selection of the *rbcL* gene may be a common phenomenon in land plants (Kapralov & Filatov, 2007). The *ycf2* gene, as a giant reading frame, possesses three sites under positive selection, and its function is still unknown (Sobanski et al., 2019). The *accD* gene encodes the *β*-carboxyl transferase subunit of acetyl-CoA carboxylase, which acetyl-CoA carboxylase catalyses the first and rate-limiting step of lipid biosynthesis (Sobanski et al., 2019). Two positively selected sites were identified in the *accD* gene. It is believed that these positively selected genes play a key role in adapting to different environments in plant evolutionary processes. In addition, the wide pantropical distributions and heterogeneous habitats might have increased the rates of evolution and speciation of *Dalbergia* species for greater adaptation (Hu et al., 2015).

### Phylogenetic relationship

We reconstructed a phylogenetic tree using the ML method and MP method based on 77 common genes of Papilionoideae species. The inferred tree has four clear clades and was consistent with previous reports (Zhang et al., 2020; Legume Phylogeny Working Group (LPWG), 2017; Zhao et al., 2021), and the phylogenetic relationships of some *Dalbergia* species were consistent with those of previous studies (Vatanparast et al., 2013; Cui, 2014). In our study, all *Dalbergia* species were divided into two main clades; one clade contained seven species (*D. hupeana*, *D. balansae*, *D. obtusifolia*, *etc.*), and most of these species were diadelphous and widely distributed in southeast Asia and southern China. The other clade contained 14 species, which could be grouped into several subclades. *D. hancei*, *D. mimosoides* and *D. cultara* were placed into one subclade with a higher bootstrap percentage. The topology structure of our phylogenetic tree was in accordance with previous phylogenetic relationships approximately. However, there are different and new advances of phylogenetic tree reconstruction. *D. obtusifolia* is a tree species and distributed in the Southwest and South Yunnan in China. The species was located at different positions in phylogenetic trees based on chloroplast DNA, nuclear DNA and their combined sequences (Cui, 2014). Our results supported the species was clustered together with *D. cochinchinensis*, and further constituted a large subclade combing *D. barienesis*, *D. oliveri*, *D. hainanensis*, *D. balansae* and *D. hupeana*. These *Dalbergia* species distributed continuously from a tropical area of Indochina peninsula to subtropical area of East Asia. The *Dalbergia* branch had a strongly supported topology and showed that some species have a close evolutionary relationship, *e.g.*, *D. odorifera* and *D. tonkinensis* and *D. hupeana* and *D. balansae.* However, species delimitation is an interesting issue that has always attracted renewed attention. *D. barienesis* has been treated as a synonym of *D. oliveri*, but the genetic divergence of its cp genomes was higher than those of two species (*D. hupeana* and *D. balansae*). Therefore, we still need further genome-wide knowledge, especially to understand the process of speciation for relatives in the *Dalbergia* genus.

## Conclusions

Herein, little difference was found in the genome size of the six sequenced cp genomes of *Dalbergia* spp. The gene content was relatively conserved, while IR boundary was highly variable in Dalbergieae species. Meanwhile, a number of cpSSRs and hotspots of nucleotides variation were screened, and selective pressure and RNA editing site of chloroplast genes also were identified in *Dalbergia* cp genomes. This will promote our understanding of their genetic variation features. Besides that, the reconstruction phylogenetic framework of chloroplast genome elucidated the relationships among species in the subfamily of Papilionoideae (Fabaceae). It also supported and improved a previous phylogenetic framework of *Dalbergia* genus based on chloroplast and nuclear DNA sequences. This indicated phylogenomic framework based on cp genome has advantages in inferring phylogenetic relationships of plants.

## Supplemental Information

10.7717/peerj.13570/supp-1 Supplemental Information 1 Supplemental Tables

10.7717/peerj.13570/supp-2 Supplemental Information 2 The raw data of cp genomes

## Additional Information and Declarations

### DNA Deposition

The following information was supplied regarding the deposition of DNA sequences: GenBank accession No. MN714219–MN714222, MN905599 and MN833948. Sequences will be made public at acceptance and are also uploaded for review.

### Data Deposition

The following information was supplied regarding data availability: The raw data are available in the Supplementary File.
